# Predictive value of high-sensitivity troponin I and D-dimer assays for adverse outcome in patients with acute pulmonary embolism

**DOI:** 10.3892/etm.2012.825

**Published:** 2012-11-22

**Authors:** THOMAS WALTER, PAUL APFALTRER, FRANK WEILBACHER, MATHIAS MEYER, STEFAN O. SCHOENBERG, CHRISTIAN FINK, JOACHIM GRUETTNER

**Affiliations:** 1 Emergency Department; University Medical Center Mannheim, Medical Faculty Mannheim, Heidelberg University, Mannheim D-68167, Germany; 2 Department of Clinical Radiology and Nuclear Medicine, University Medical Center Mannheim, Medical Faculty Mannheim, Heidelberg University, Mannheim D-68167, Germany

**Keywords:** pulmonary embolism, troponin I, D-dimer, adverse clinical outcome

## Abstract

High-sensitivity troponin (hs-cTn) assays enable the troponin cutoff value to be lowered, resulting in an increase of sensitivity at the cost of specificity. In the present study, the risk of a short-term adverse outcome was assessed in patients with acute pulmonary embolism (PE) using high-sensitivity troponin I (hs-cTnI). We used a cutoff value of 0.1 ng/ml in accordance with current guidelines for unstable angina (UA)/non-ST-segment elevation myocardial infarction (NSTEMI), although the detection limit of the troponin assay is lower. In addition, the risk of an adverse outcome in patients with acute PE was investigated with respect to initial D-dimer serum concentrations. In 65 patients with confirmed acute PE, hs-cTnI and D-dimer values were measured. Adverse clinical outcome was defined as cardiogenic shock, cardiopulmonary resuscitation, mechanical ventilation, vasopressor therapy, thrombolysis, catheter intervention or mortality within 60 days of PE. Patients with acute PE and serum hs-cTnI values >0.1 ng/ml showed significantly higher D-dimer concentrations (P= 0.0467) and a 5-fold increased risk of an adverse clinical outcome [odds ratio (OR), 4.9; 95% confidence interval (CI), 1.28–18.66; P=0.0235] compared with patients with acute PE and hs-cTnI values <0.1 ng/ml. In patients with acute PE suffering from adverse clinical outcome, D-dimer concentrations were significantly elevated compared with those in patients with acute PE without adverse clinical outcome (P=0.02). In patients with acute PE, a hs-cTnI cutoff value of 0.1 ng/ml, which is identical to the recommended cutoff value of NSTEMI, may identify patients with a 5-fold increased risk of a short-term adverse outcome. D-dimer values are significantly higher in PE patients with elevated hs-cTnI values as well as in patients with an adverse outcome.

## Introduction

Acute pulmonary embolism (PE) is a common cardiovascular emergency with a wide spectrum of clinical presentations. Strategies for risk stratification in patients with acute PE are crucial so that a risk-adjusted management stategy may be adapted for these patients with regard to clinical outcome (1,2). In the current guidelines (1), risk markers useful for risk stratification in PE are classified into three groups. Clinical assessment allows stratification into high-risk and non-high-risk PE. Non-high-risk PE may be further stratified according the presence of markers of right ventricular (RV) dysfunction and/or myocardial injury (determined by troponin I or T serum concentrations) into intermediate- and low-risk PE (1). Recently, new generations of high-sensitivity troponin assays have been developed for measuring troponin levels that would register as ‘zero’ or considered to be in the ‘normal’ range with conventional assays (3,4). Numerous studies have demonstrated that elevated cardiac troponin levels are associated with a high risk of short-term adverse outcome in patients presenting with acute PE (5–7). It has been shown that a high-sensitivity troponin (hs-cTn) T assay cannot be used alone to diagnose PE (3). Studies using a high-sensitivity troponin assay showed a 100% sensitivity and negative predictive value with regard to the 30-day risk of mortality or major complications in stable patients with acute PE (6,8). However, for the clinician the heterogeneity of troponin assays and their lack of harmonization continue to result in analytical and interpretative challenges (9). In the current guidelines for unstable angina (UA)/non-ST-segment elevation myocardial infarction (NSTEMI), a troponin elevation >0.1 ng/ml has been defined as a predictor of high-risk of mortality or non-fatal myocardial infarction in patients with non-ST-segment-elevation acute coronary syndrome (NSTE-ACS) (10).

Plasma D-dimer has been widely recognized to be useful in the diagnostic work-up of PE (11–17). D-dimer, a degradation product of crosslinked fibrin, is elevated in plasma in the presence of an acute clot due to simultaneous activation of coagulation and fibrinolysis (1,15,16,18). The negative predictive value of D-dimer is high, but the positive predictive value is low (1,12–14,16,19,20). Particularly elevated D-dimer levels in patients with acute PE may even be the result of the acute thromboembolic event and comorbidities (11,14,16–18). Therefore, the level of D-dimer may be extremely high as a result of concomitant cancer or chronic obstructive pulmonary disease (COPD) (11,14,16). These conditions are known predictors of outcome in patients with acute PE (11,18). The correlation between D-dimer levels and the burden of PE as assessed by CT angiography has been evaluated in several studies, but the association between troponin and D-dimer levels remains controversial (11).

The aim of the study was to assess the risk of an adverse clinical outcome in patients with PE using an hs-cTnI assay but defining a ‘higher’ troponin I cutoff value of 0.1 ng/ml in accordance to current guidelines of UA/NSTEMI (10). Additionally, the risk of an adverse outcome in patients with acute PE was investigated with respect to their initial D-dimer serum concentrations.

## Materials and methods

### Study population

In a retrospective analysis, our institution’s radiology information system (RIS) was searched for patients who were referred by the Chest Pain Unit of the University Medical Center Mannheim, Heidelberg University (Mannheim, Germany) for CT-angiography of the pulmonary artery (CTPA) due to clinical suspicion of PE, and had received hs-cTnI and D-dimer serum assays. Between March 1 and November 30, 2011, 210 patients were identified and further analyzed by two observers for the presence of acute PE.

In total we identified 65 patients (mean age 67±11 years; range 20–90; 36 females, 29 males) with immediate hs-cTnI and D-dimer serum level measurements during admission and instant confirmation of PE with CTPA. Due to the retrospective nature of the study protocol, the institutional ethical review board waived the need for informed consent.

### Laboratory measurements

hs-cTnI and D-dimer serum levels were quantified from a venous blood sample, which was drawn in the hospital’s certified chest pain unit immediately after admission. hs-cTnI levels were measured with a LOCI immunoassay (Vista; Siemens Healthcare Diagnostics, Eschborn, Germany). We used a cutoff value of 0.1 ng/ml for the evaluation of ACS in patients with acute chest pain in accordance with the American College of Cardiology/American Heart Association (ACC/AHA) guidelines of UA/NSTEMI (10). D-dimer values were determined with the Tina-quant D-dimer assay from Roche Diagnostics (Mannheim, Germany).

### Clinical outcome

We defined adverse clinical outcome as cardiogenic shock (systolic blood pressure <90 mmHg; heart rate >100 bpm), cardiopulmonary resuscitation, mechanical ventilation, vasopressor therapy, thrombolysis, catheter intervention or mortality within 60 days of PE. For the determination of outcome, the hospital records of all 65 patients were reviewed.

### Statistical analysis

Statistical analysis was performed using JMP 9.0 (SAS Institute, Cary, NC, USA). Continuous variables are expressed as means ± SD. The Shapiro-Wilk test was applied to determine probability distribution. The Mann-Whitney U test was used if the data were not normally distributed. To assess the dependence between the tested variables, multivariate linear regression was performed with backward stepwise elimination of least and non-significant parameters. A two-tailed P<0.05 was considered to indicate a statistically significant result.

## Results

Patient baseline characteristics are summarized in Table I. In the current study, 65 patients with acute PE were enrolled of whom 12 (19%) suffered from adverse clinical outcome within 60 days. Due to worsening vital signs, 11 patients required transfer to the intermediate care unit (ICU). In these cases 3 (27%) required mechanical ventilation and 2 (18%) catecholamine treatment. Two patients succumbed within the 60 day follow-up, but the mortalities were not directly associated with PE (stroke, n=2).

**Figure 1.**
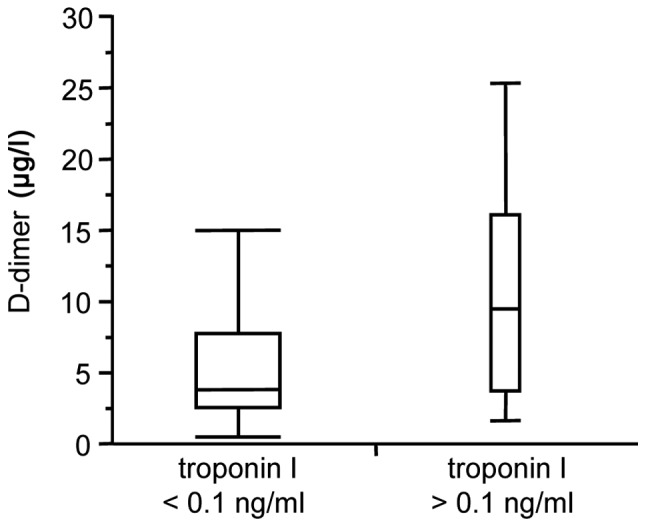
D-dimer levels in patients with acute pulmonary embolism with high-sensitivity troponin I (hs-cTnI) values <0.1 ng/ml (n=49) compared with those patients with hs-cTnI values >0.1 ng/ml (n=16). Data are expressed as medians with 25th and 75th percentiles (boxes) and 1st and 99th percentiles (whiskers).

For statistical analysis we defined a hs-cTnI cutoff value of 0.1 ng/ml according to the cutoff value we routinely use (in the context of other clinical findings) in our chest pain unit for the clinical evaluation of NSTE-ACS in patients with acute chest pain. In the present study, patients with serum hs-cTnI values >0.1 ng/ml showed significantly higher D-dimer concentrations than patients with acute PE and hs-cTnI levels <0.1 ng/ml (P=0.0467; Fig. 1). In patients with acute PE and hs-cTnI values >0.1 ng/ml, the risk of an adverse clinical outcome in the next 60 days was increased 5-fold compared with that in patients with acute PE and hs-cTnI values <0.1 ng/ml [odds ratio (OR), 4.9; 95% confidence interval (CI), 1.28–18.66; P= 0.0235]. In addition, in patients with acute PE suffering from adverse clinical outcome, significantly elevated D-dimer concentrations were measured compared with those in patients with acute PE without adverse clinical outcome (P=0.02; Table II).

## Discussion

European (1) and American guidelines (21) emphasize the role of estimating the likelihood of PE and the risk of early mortality due to PE, which is crucial for the selection of appropriate treatment (22). Myocardial injury in patients with PE may be detected by troponin I or T testing. Positive results are associated with an intermediate risk of short-term mortality in acute PE (1).

The reason for the release of troponins in a subset of patients with acute PE remains unclear (7). However, explanations include hypoxemia due to perfusion-ventilation mismatch, hypoperfusion as a consequence of low output and reduced coronary blood flow, as well as cell injury caused by acute dilation of the right ventricle, or a combination of these factors (7,23). To date, it is still unclear if positive troponin values in hemodynamically stable patients identify those who would benefit from thrombolysis (7,23).

The role of elevated troponins I and T, which are correlated with in-hospital mortality and a complicated clinical course in patients with acute PE, is reviewed in the European Society of Cardiology (ESC) guidelines on the diagnosis and management of acute PE (1), and numerous other studies (5–8,23,24). In these previous studies, different troponin assays and cutoff values for troponins were used. Conventional troponin assays are characterized by inadequate precision at the lower detection limit (6). High-sensitivity and high-sensitivity troponin assays have been developed and are already widely used in clinical practice in order to overcome this limitation. Lankeit *et al*(6,8) assessed the role of cardiac troponin T in the risk assessment of normotensive patients with acute PE using a high-sensitivity assay. They defined a high-sensitivity TnT cutoff (14 pg/ml) showing a 100% sensitivity and negative predictive value with regard to the 30-day risk of mortality or major complications (6,8). This may be helpful for risk stratification in patients with documented PE and it may aid the identification of candidates for early discharge and home treatment, but for the clinician in the emergency department the heterogeneity of cardiac troponin assays and their lack of harmonization continue to result in analytical and interpretative challenges. Numerous asymptomatic patients routinely present in the clinic with elevated troponin values and the interpretation of these results becomes more difficult the lower the cutoffs of the new high-sensitivity assays were defined.

At present, troponin levels at the recommended cutoff may be measured in >80% of healthy individuals with a coefficient of variation <10% (4). The use of hs-cTn assays may lower the troponin cutoff value for diagnosis of acute myocardial infarction (AMI) in accordance with the current definitions (9,10,25,26), resulting in a 160% increase of chest pain patients diagnosed with AMI and a 200% increase of troponin-positive patients with non-coronary cardiac chest pain (4). To date it is not clear whether the new hs-cTn methods will lead to more clarity or confusion in routine clinical practice. The introduction of hs-cTn assays increases the sensitivity for identifying patients with myocardial injury, even at the time of initial presentation in the emergency department, at the cost of specificity. On the other hand, even small increases of cTnI levels in patients presenting with acute chest pain to the emergency department are indicative of an adverse short- and long-term prognosis (4).

In the present study, the determination of troponin I was performed using a high-sensitivity assay. Although the detection limit of the troponin assay we used is much lower, we defined a hs-cTnI cutoff of 0.1 ng/ml for statistical analysis in patients with acute PE in the present study, in accordance with current guidelines for UA/NSTEMI (10). To assess the risk of adverse clinical outcome, patients with elevated (>0.1 ng/ml) and not elevated (<0.1 ng/ml) troponin I values were compared. Our data demonstrate that patients with an acute PE and hs-cTnI concentrations >0.1 ng/ml have a 5-fold increased risk of an adverse clinical outcome in the next 60 days. In a meta-analysis by Becattini *et al*(5), a positive troponin T assay during acute PE was associated with an OR for mortality of 7.95 (95% CI, 3.79–16.65) during short-term follow-up, but in the studies analyzed, the definition of a positive troponin assay varied. In a recent study a concentration-dependent association between troponin T and outcome was observed (24). Using high-sensitivity troponin assays would aid identification of ‘very low risk’ patients in whom extremely favourable outcomes may be expected. However, the best treatment strategy for hemodynamically stable patients with acute PE and very low ‘positive’ troponin values remains unresolved.

Patients with acute PE presenting with elevated troponin values have a higher risk for future adverse clinical outcome, but for clinical practice it is not clear yet whether patients with acute PE and very low troponin values require more intensive monitoring (e.g. in the intermediate care unit). In addition, there are no therapeutic consequences for these patients thus far. Thus, our data demonstrate that defining a cutoff troponin value of 0.1 ng/ml, which is in accordance with current guidelines for UA/NSTEMI (10), detects patients with acute PE with a 5-fold higher risk of an adverse clinical outcome. This cutoff may be used to identify patients who should be monitored in the intermediate care unit during the initial treatment period to identify relatively frequent occurrences of clinical deterioration and the need for rapid treatment escalation. However, further studies are required to confirm this hypothesis. In the emergency department, the interpretation of clinical presentation and clinical information is important in decision-making for patients presenting with acute PE and positive troponin values.

To select a suitable diagnostic strategy for hemodynamically stable patients prior to laboratory tests or imaging, the assessment of clinical probability of PE is recommended (1,2,17,20). The most frequently used clinical prediction rules for PE are the Wells score (27) and the revised Geneva score (28). Measurement of D-dimer is not useful for confirming PE (1,20). The negative predictive value of D-dimer is high, but the positive predictive value is low (1,12–14,16,19,20). Normal D-dimer values may be used to exclude PE in patients with either a low or a moderate probability of PE (1,13,15–17,20). In patients admitted to the emergency department, plasma D-dimer measurement combined with clinical probability assessment allows PE to be ruled out in approximately 30% of patients, with a 3-month thromboembolic risk <1% in patients left untreated (1,20). Current guidelines recommend multi-detector CT (MDCT) as the second-line test in patients with an elevated D-dimer level and as the first-line test in patients with a high clinical probability of acute PE (1). However, false-negative results of MDCT have been reported in patients with a high clinical probability of PE (1,29). This situation is infrequent and the 3-month thromboembolic risk is low in such patients (1,13,30). In patients with high D-dimer levels and negative MDCT results the incidence of thromboembolic events is 1.5% compared witĥ0.5% in patients with a normal D-dimer level (2,31). Therefore, the necessity of performing further tests and the nature of these tests in such patients is controversial (1). In the present study, we demonstrated that patients with acute PE and adverse clinical outcome have significantly higher D-dimer serum concentrations than patients with acute PE without adverse clinical outcome. This is in accordance with a meta-analysis by Becattini *et al*(11) which suggests that high D-dimer levels are associated with short-term and 3-month mortality rates in patients with acute PE. Therefore, although the positive predictive value of D-dimer is low, measurement of D-dimer concentrations in patients with a high clinical probability of PE and negative MDCT result may be helpful to evaluate the risk of a further adverse outcome in these patients and the need for treatment. Normal or ‘low elevated’ D-dimer levels in these patients may be used to identify patients with a good prognosis and inform the decision to leave patients untreated. To confirm this hypothesis larger prospective investigations, with additional identification of a threshold value of D-dimer that defines increased risk of an adverse outcome in patients with acute PE, are required.

The correlation between D-dimer levels and the burden of PE as assessed by CT angiography has been evaluated in several studies, but the association between troponin and D-dimer levels remains controversial (11). In the present study, we observed significantly elevated D-dimer concentrations in patients with acute PE and hs-cTnI values >0.1 ng/ml, compared with those in patients with acute PE and hs-cTnI values <0.1 ng/ml. This demonstrates that patients with signs of myocardial injury due to acute PE had significantly higher D-dimer serum levels.

In conclusion, in patients with acute PE, a hs-cTnI cutoff value of 0.1 ng/ml, which is identical to the recommended cutoff value of NSTEMI, may identify patients who have a 5-fold increased risk of a short-term adverse outcome. D-dimer values were significantly higher in PE patients with hs-cTnI values >0.1 ng/ml as well as in patients with an adverse outcome.

## Figures and Tables

**Table I.** Baseline characteristics of 65 patients suffering from acute pulmonary embolism.

Characteristic	All patients (n=65)
Age, years	
Mean ± SD	67±11
Range	20–90
Gender, n (%)	
Male	29 (45)
Female	36 (55)
Medical history, n (%)	
Thrombosis and/or pulmonary embolism	10 (15)
Cancer	28 (43)
Coronary artery disease	6 (9)
Congestive heart failure	4 (6)
COPD	11 (17)
Renal failure	6 (9)
Heart rate, bpm	84
Systolic blood pressure, mmHg	128
Oxygen saturation, %	96
Adverse clinical outcome within 60 days, n (%)	12 (19)
Transfer to ICU	11 (17)
Mechanical ventilation	3 (5)
Catecholamine treatment	2 (3)
Mortality	2 (3)
Thrombolysis	1 (1)
Baseline laboratory values, n (%)	
hs-cTnI values	
>0.015 ng/ml	24 (37)
0.015–0.1 ng/ml	8 (12)
<0.1 ng/ml	16 (25)
D-dimer >0.5 mg/l	65 (100)

COPD, chronic obstructive pulmonary disease; ICU, intermediate care unit; hs-cTnI, high-sensitivity troponin I.

**Table II.** D-dimer concentrations in patients with acute pulmonary embolism in terms of hs-cTnI values and adverse clinical outcome within the next 60 days.

Variable	D-dimer (μg/l)	P-value
High-sensitivity troponin I values		
<0.1 ng/ml	8.1 ± 8.9	0.0467
>0.1 ng/ml	12.0 ± 9.2	
Adverse clinical outcome within 60 days		
No	6.8 ± 6.8	0.02
Yes	12.1 ± 8.7	

hs-cTnI, high-sensitivity troponin I.

## References

[1] Torbicki A, Perrier A, Konstantinides S (2008). Guidelines on the diagnosis and management of acute pulmonary embolism: the Task Force for the Diagnosis and Management of Acute Pulmonary Embolism of the European Society of Cardiology (ESC). Eur Heart J.

[2] Agnelli G, Becattini C (2010). Acute pulmonary embolism. N Engl J Med.

[3] Hogg K, Haslam S, Hinchliffe E, Sellar L, Lecky F, Cruickshank K (2011). Does high-sensitivity troponin measurement aid in the diagnosis of pulmonary embolism?. J Thromb Haemost.

[4] Christ M, Bertsch T, Popp S, Bahrmann P, Heppner HJ, Muller C (2011). High-sensitivity troponin assays in the evaluation of patients with acute chest pain in the emergency department. Clin Chem Lab Med.

[5] Becattini C, Vedovati MC, Agnelli G (2007). Prognostic value of troponins in acute pulmonary embolism: a meta-analysis. Circulation.

[6] Lankeit M, Friesen D, Aschoff J (2010). Highly sensitive troponin T assay in normotensive patients with acute pulmonary embolism. Eur Heart J.

[7] Janata KM, Leitner JM, Holzer-Richling N, Janata A, Laggner AN, Jilma B (2009). Troponin T predicts in-hospital and 1-year mortality in patients with pulmonary embolism. Eur Respir J.

[8] Lankeit M, Jimenez D, Kostrubiec M (2011). Predictive value of the high-sensitivity troponin T assay and the simplified pulmonary embolism severity index in hemodynamically stable patients with acute pulmonary embolism: a prospective validation study. Circulation.

[9] Thygesen K, Mair J, Katus H (2010). Recommendations for the use of cardiac troponin measurement in acute cardiac care. Eur Heart J.

[10] Pollack CV, Braunwald E (2008). 2007 update to the ACC/AHA guidelines for the management of patients with unstable angina and non-ST-segment elevation myocardial infarction: implications for emergency department practice. Ann Emerg Med.

[11] Becattini C, Lignani A, Masotti L, Forte MB, Agnelli G (2012). D-dimer for risk stratification in patients with acute pulmonary embolism. J Thromb Thrombolysis.

[12] Goldhaber SZ, Simons GR, Elliott CG (1993). Quantitative plasma D-dimer levels among patients undergoing pulmonary angiography for suspected pulmonary embolism. JAMA.

[13] Abcarian PW, Sweet JD, Watabe JT, Yoon HC (2004). Role of a quantitative D-dimer assay in determining the need for CT angiography of acute pulmonary embolism. AJR Am J Roentgenol.

[14] Kabrhel C, Mark Courtney D, Camargo CA (2010). Factors associated with positive D-dimer results in patients evaluated for pulmonary embolism. Acad Emerg Med.

[15] Kearon C, Ginsberg JS, Douketis J (2006). An evaluation of D-dimer in the diagnosis of pulmonary embolism: a randomized trial. Ann Intern Med.

[16] Righini M, Perrier A, De Moerloose P, Bounameaux H (2008). D-Dimer for venous thromboembolism diagnosis: 20 years later. J Thromb Haemost.

[17] Yin F, Wilson T, Della Fave A (2012). Inappropriate use of D-dimer assay and pulmonary CT angiography in the evaluation of suspected acute pulmonary embolism. Am J Med Qual.

[18] Goldhaber SZ (2004). Pulmonary embolism. Lancet.

[19] Dunn KL, Wolf JP, Dorfman DM, Fitzpatrick P, Baker JL, Goldhaber SZ (2002). Normal D-dimer levels in emergency department patients suspected of acute pulmonary embolism. J Am Coll Cardiol.

[20] Gupta RT, Kakarla RK, Kirshenbaum KJ, Tapson VF (2009). D-dimers and efficacy of clinical risk estimation algorithms: sensitivity in evaluation of acute pulmonary embolism. AJR Am J Roentgenol.

[21] Jaff MR, McMurtry MS, Archer SL (2011). Management of massive and submassive pulmonary embolism, iliofemoral deep vein thrombosis, and chronic thromboembolic pulmonary hypertension: a scientific statement from the American Heart Association. Circulation.

[22] Labyk A, Ciurzynski M, Jankowski K (2012). Acute pulmonary embolism: analysis of consecutive 353 patients hospitalised in a single centre. A 3-year experience. Kardiol Pol.

[23] Horlander KT, Leeper KV (2003). Troponin levels as a guide to treatment of pulmonary embolism. Curr Opin Pulm Med.

[24] Ng AC, Yong AS, Chow V, Chung T, Freedman SB, Kritharides L (2011). Cardiac troponin-T and the prediction of acute and long-term mortality after acute pulmonary embolism. Int J Cardiol.

[25] Thygesen K, Alpert JS, White HD (2007). Universal definition of myocardial infarction. J Am Coll Cardiol.

[26] Alpert JS, Thygesen K, Jaffe A, White HD (2008). The universal definition of myocardial infarction: a consensus document: ischaemic heart disease. Heart.

[27] Wells PS, Anderson DR, Rodger M (2000). Derivation of a simple clinical model to categorize patients probability of pulmonary embolism: increasing the models utility with the SimpliRED D-dimer. Thromb Haemost.

[28] Le Gal G, Righini M, Roy PM (2006). Prediction of pulmonary embolism in the emergency department: the revised Geneva score. Ann Intern Med.

[29] Stein PD, Fowler SE, Goodman LR (2006). Multidetector computed tomography for acute pulmonary embolism. N Engl J Med.

[30] van Belle A, Buller HR, Huisman MV (2006). Effectiveness of managing suspected pulmonary embolism using an algorithm combining clinical probability, D-dimer testing, and computed tomography. JAMA.

[31] Perrier A, Roy PM, Sanchez O (2005). Multidetector-row computed tomography in suspected pulmonary embolism. N Engl J Med.

